# Implications of a conserved region of bluetongue virus protein VP2 in cross-neutralisation of bluetongue virus serotypes

**DOI:** 10.4102/ojvr.v87i1.1816

**Published:** 2020-10-08

**Authors:** Shiva J. Jyothi, Sunil R. Patil, Narasimha Y. Reddy, Rao P. Panduranga, Uma Madala, Gnana M. Prakash, Kalyani Putty

**Affiliations:** 1 Department of Veterinary Biotechnology, College of Veterinary Science, P.V. Narsimha Rao Telangana Veterinary University, Hyderabad, India; 2 Biovet Pvt Ltd., Malur, Karnataka, India; 3 Ella Foundation, Turkapally, Hyderabad, India; 4 Department of Animal Genetics and Breeding, College of Veterinary Science, P.V. Narsimha Rao Telangana Veterinary University, Hyderabad, India

**Keywords:** bluetongue, subunit vaccine, conserved VP2, cross-neutralisation, broad-spectrum protection

## Abstract

Bluetongue (BT) is a vector-borne disease of ruminants caused by Bluetongue virus (BTV). Twenty-nine different serotypes of BTV are currently reported throughout the world. The main objective of this study is the development of a subunit vaccine model that could potentially be adapted to provide broad spectrum protection against multiple BTV serotypes, which the conventional vaccines fail to address. To this end, three different BTV proteins (conserved region of viral protein [VP]2, VP5 and NS1) were expressed and purified in an *Escherichia coli* expression system. The immunogenicity of these proteins was tested in murine models using the Montanide^TM^ ISA 201 VG adjuvant. BALB/c mice were immunised thrice (with individual proteins and a mixture of three proteins) at two-week intervals and were monitored until Day 40 post-infection/vaccination. Protein-specific antibodies directed against the recombinant proteins were detected by indirect enzyme-linked immunosorbent assay. Neutralising antibody (Nab) titres and cross-neutralisation against a range of BTV serotypes (BTV-1, -2, -4, -5, -9, -10, -12, -16, -21, -23 and -24) were determined by serum neutralisation test. The recombinant proteins elicited higher Nab titres compared with the inactivated vaccine group, except for BTV-1, where the inactivated vaccine group elicited higher Nab titres. Additive effect of the three proteins was not observed as the Nab titres generated with a combination of conserved VP2, VP5 and NS1 was similar to those of the individual protein groups. Whilst BTV-12 could only be neutralised by serum raised against the inactivated vaccine group, BTV-5 and -24 could not be neutralised by any of the groups tested. Our cumulative data suggest that the conserved regions of VP2 (cVP2), VP5 and NS1 could play an important part in the novel vaccine design against multiple BTV serotypes. Importantly, given that VP2 was already known to elicit a serotype-specific immune response against BT, we report, for the first time, that the conserved region of VP2 has the ability to induce cross-protective immune response.

## Introduction

Bluetongue virus (BTV) is a member of the family *Reoviridae* and is the causative agent of bluetongue (BT) disease, a haemorrhagic disease of sheep and some species of wild ruminants (Mertens & Diprose 2004). So far, 29 serotypes (Kartika Lakshmi et al. 2018; Maan et al. 2016) of BTV have been identified worldwide and 24 serotypes have been reported to circulate in India (Hemadri et al. 2017; Krishnajyothi et al. 2016). As per a recent study conducted during 2014–2018 by our group, BTV serotypes BTV-1, -2, -4, -9, -12, -16, -21 and -24 seemed to be the circulating serotypes in India (personal observation, unpublished data). An inactivated pentavalent vaccine consisting of BTV serotypes BTV-1, -2, -10, -16 and -23 is currently used in some parts of India. However, circulation of multiple serotypes, often within the same animal, and little or no cross-protection between serotypes makes BTV control and prevention a challenge (Hemadri et al. 2017). Bluetongue virus has a double-stranded RNA (dsRNA) genome composed of 10 linear segments encoding seven structural proteins (viral protein [VP]1–VP7) and five non-structural proteins (NS1, NS2, NS3a, NS3b and NS4) (Rao et al. 2017). VP2, a major viral serotype determinant, is responsible for virus attachment and haemagglutination. VP5, coded by segment 6, induces membrane permeabilisation during onset of the infection and can cause syncytia formation, affect specificity of virus neutralisation and also show partial correlation with virus serotype. NS1 is a viral protein translation enhancer that co-localises with the centrosome and may play a role in disrupting and blocking cell division in mammalian cells, and it is highly conserved among different BTV serotypes. It contains epitopes associated with both T-cell and humoral responses, and antibody responses against NS1 protein may be important contributors to immune protection (Anderson, Hagglund & Breard; 2014; Rao et al. 2017). Protein-based vaccines for BT were based on the initial observation that VP2, in combination with VP5, elicits protective immunity in vaccinated sheep, following which several studies reported potential vaccines for other immunogenic proteins of BTV including NS1 (Anderson, Hagglund & Breard 2013, 2014; Jones et al. 1996; Marin-Lopez et al. 2014; Mohd Jaffar et al. 2014; Roy et al. 1990). VP2 is the most variable protein unique to each BTV serotype; sequence data indicate that the most conserved region of VP2 (cVP2) between serotypes was evident from 338 to 383 amino acids predicted with two major histocompatibility complex class I (MHCI) and two major histocompatibility complex class II (MHCII) binding sites and three B-cell epitopes, suggesting cVP2 has a strong immunogenicity potential (Shiva Jyothi et al. 2018). Conventional vaccines, though efficient, are not without disadvantages. Incomplete attenuation or inactivation, ability of vaccine strains to reassort with field strains, adverse clinical reactions to vaccination and emergence of new serotypes limit the use of ‘serotype-specific protection conferring’ conventional vaccines. With this perspective, the main objective of this study was to develop a broad-spectrum subunit vaccine model for BT that can confer protection against a range of BTV serotypes. Here, cVP2, VP5 and NS1 proteins were expressed in *Escherichia coli*. Immunogenicity potential of these proteins was tested in BALB/c murine models and compared with the conventional inactivated pentavalent vaccines. Finally, the cross-neutralisation potential of the proposed vaccine model was evaluated and discussed.

## Materials and methods

### Virus and cell lines

Bluetongue virus serotypes BTV-1, -2, -4, -5, -9, -10, -12, -16, -21, -23 and -24 isolated during BT outbreaks in 2011–2016 were plaque purified and their identities were confirmed by real-time polymerase chain reaction (Maan et al. 2016). The virus was propagated in BHK-21, and Vero cell lines were grown in minimal essential media (Gibco, United States [US]) supplemented with 10% foetal bovine serum (Gibco, US) at 37 °C in an atmosphere containing 5% CO_2_ (World Organisation for Animal Health 2012).

### Generation of recombinant plasmids

The conserved region of VP2 (234 bp; nucleotide positions: 1012–1246, GenBank accession: KP339225) was amplified from BTV-16 complementary DNA using primers F (*Bam*HI) 5’*GGATCCg*ATGCGTTTCTATGTGTTGCTAAT3’ and R (*Eco*RI) 5’*GAATTC*TCAGTCAAAGAGGTTAACGCGCC3’ and cloned into expression vector pRSET-B (Invitrogen, US). For VP5 and NS1, codon optimised artificially synthesised BTV-16 full length genes (GenBank accession numbers: KF664138 and KF387525 for VP5 and NS1, respectively) were cloned in pET-28b (+) (Invitrogen, US).

### Protein expression by autoinduction and purification

The constructed plasmids were transformed into *E. coli* BL21 (DE3) competent cells (Sigma, US) according to the manufacturer’s protocol and previously described procedure (Hanahan et al. 1991). Protein expression by autoinduction, as described in an earlier study, was followed for all three proteins (Studier 2005). The proteins were then subjected to sodium dodecyl sulfate polyacrylamide gel electrophoresis (SDS-PAGE) and Western blotting (Shi & Jackowski, 1998) to detect His-tagged cVP2 (9.35 KDa), VP5 (59.8 KDa) and NS1 (61.2 KDa). Protein purification was done using nickel (Ni-NTA) affinity chromatography (Qiagen, US) as per the manufacturer’s instructions. Refolding of proteins purified under denaturing conditions was done as mentioned in a previous study (Anderson et al. 2014). Final confirmation of the eluted proteins was done using SDS-PAGE along with Western blotting.

### Mice immunisation

Thirty-six (6- to 8-week-old) female BALB/c mice were housed at the animal house of the College of Veterinary Science, Hyderabad. They were divided into six groups (Groups 1–6) of six mice each; Groups 1–4 were injected subcutaneously on Days 0, 14 and 28 with 25 *µ*g of each protein in Montanide^TM^ ISA 201 VG emulsion (Table 1). The commercially available inactivated pentavalent vaccine (RAKSHA-BLU, Indian Immunologicals, India) containing BTV-1, -2, -10, -16 and -23 was used to immunise the Group 5 mice. Six non-immunised BALB/c mice administered with Montanide^TM^ ISA 201 VG in phosphate-buffered saline were used as the control group (Group 6). All the animals in this study were examined daily for the presence of reactions, if any, until Day 40 and sacrificed; blood was then collected by heart puncture for serological analyses.

**TABLE 1:** Protocol of mice immunisation.

Group	No. of animals	Day 0	Day 14	Day 28	Day 41
Group 1 (cVP2)	6	25 *µ*g	25 *µ*g	25 *µ*g	Blood collection
Group 2 (VP5)	6	25 *µ*g	25 *µ*g	25 *µ*g	Blood collection
Group 3 (NS1)	6	25 *µ*g	25 *µ*g	25 *µ*g	Blood collection
Group 4 (cVP2+VP5+NS1)	6	25 *µ*g each	25 *µ*g each	25 *µ*g each	Blood collection
Group 5 (inactivated pentavalent vaccine)	6	100 *µ*L	100 *µ*L	100 *µ*L	Blood collection
Group 6 (Control)	6	Montanide	Montanide	Montanide	Blood collection

VP, viral protein.

### Antibody detection (indirect enzyme-linked immunosorbent assay and serum neutralisation assay)

Antibody response against specific proteins was monitored by indirect enzyme-linked immunosorbent assay (ELISA) using 1/200 dilution of collected serum samples (Anderson et al. 2014). Neutralising antibodies were determined (Savini et al. 2004). Plaque-purified BTV-1, -2, -4, -5, -9, -10, -12, -16, -21, -23 and -24 were used as antigens. Serum samples obtained two weeks after the third immunisation were serially diluted (1/10, 1/20, 1/40, 1/80, 1/160, 1/320, 1/640 and 1/1280), incubated with an equal quantity of 100 tissue culture infective dose (TCID)_50_/50 *µ*L and added to confluent monolayers of Vero cells in 96-well plates. Plates were observed under an inverted microscope at 12-h interval for cytopathic effect (CPE), and the final titre was taken on Day 5. All dilutions were performed in quadruplicate, and the assays were repeated at least three times. The neutralisation titres were determined as the highest dilution of serum giving a 50% neutralisation endpoint and expressed as a log_10_ reciprocal of the highest positive serum dilution.

### Statistical analysis

Data were presented as means and standard deviations, where applicable, and the statistical significance of differences between groups was determined as *p-*values using Student’s *t*-test in Excel. *p*-Value < 0.05 was considered to be significant.

### Ethical consideration

All the animal experimentation protocols were approved by the host institute’s Institutional Animal Ethics Committee (IAEC no. 16/6/IAEC/2017/16.05.17).

## Results and discussion

The development of effective vaccines for viruses with multiple distinct serotypes is highly challenging. Subunit vaccines address the drawbacks conventional vaccines pose and have an advantage of being differentiating infected from vaccinated animals (DIVA) compliant. Humoral response is the main component of the immune response against BTV; contribution of the cellular component, although it appears to be important, is not well understood (Anderson et al. 2013; Marin-Lopez et al. 2014). The major aim of the subunit vaccine design in this study was to determine its (1) role in humoral immune response and (2) potential to induce cross-serotype reactive immune responses.

### Recombinant bluetongue virus proteins’ expression and purification

**FIGURE 1:**
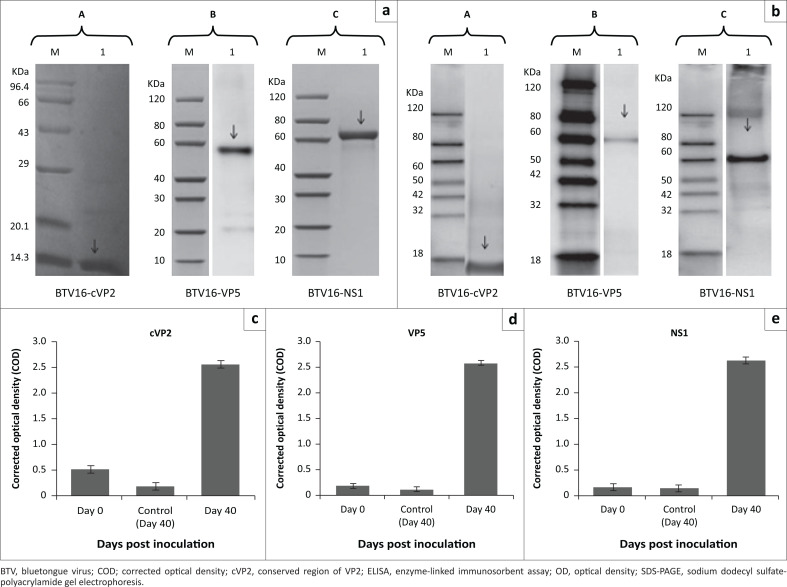
Identification of purified BTV recombinant proteins and detection of protein-specific antibodies by indirect ELISA. (a) Ni-NTA purification of recombinant BTV proteins, and SDS-PAGE analysis of purified cVP2, VP5 and NS1: arrowheads indicate recombinant BTV proteins at the expected molecular weights. A (Lane 1): cVP2, 9.35 KDa; B (Lane 1): VP5, 59.8 KDa; C (Lane 1): NS1, 61.2 KDa. Lane M: protein marker. (b) Western blot confirmation of tagged proteins: His-tagged recombinant BTV proteins were detected using anti-His antibodies. Lane M: protein marker; A (Lane 1): cVP2, 9.35 KDa; B (Lane 1): VP5, 59.8 KDa; C (Lane 1): NS1, 61.2 KDa. (c, d, e) Protein-specific antibodies against BTV cVP2, VP5 and NS1 in immunised mice. Mice were vaccinated thrice with a 2-week interval using BTV recombinant proteins (Table 1). The COD values were calculated as (COD = OD Sample – OD background) and compared with preimmunised and control group.

Conserved region of VP2 was detected in the soluble fraction of the *E. coli* cell lysate; VP5 and NS1 were detected in inclusion bodies and purified under denaturing conditions followed by refolding of the eluted proteins. Purified proteins were desalted by dialysis and their concentrations were found to be 1.8 mg/mL, 1.1 mg/mL and 0.45 mg/mL for cVP2, VP5 and NS1, respectively. Sodium dodecyl sulfate polyacrylamide gel electrophoresis gel analysis detected proteins of desired size with up to 90% purity indicated by the absence of bands of higher and lower molecular weights than the desired sizes (Figure 1a and b).

Several host systems were reported earlier in the successful expression of recombinant BTV proteins, including baculoviral, bacterial, yeast and mammalian cells (Mohd Jaffar et al. 2014). In this study, we have succeeded in making use of the *E. coli* expression system, suggesting that the BTV proteins can be expressed in simpler bacterial expression systems without the use of complex eukaryotic expression systems. Despite the difficulties in protein expression (particularly for the larger BTV proteins) often observed with the *E. coli* expression systems, such as formation of inclusion bodies, potential misfolding of proteins and lack of post-translational modifications, this system is preferred for its ease of handling and scalability of production of potential vaccine candidates. Autoinduction depends on glucose catabolite repression and lactose induction to tightly control protein expression (Studier 2005). Because of the reduced need for sample processing, higher yields of the target protein and ease of culture scale up, this system when compared with isopropyl β-d-1-thiogalactopyranoside (IPTG) induction is a very attractive method for achieving high throughput protein expression.

### Specific antibody responses to the recombinant proteins in mice

Indirect ELISA was used to evaluate specific antibody responses to cVP2, VP5 and NS1 in mice on Day 40 post-immunisation. All three proteins induced protein-specific immune responses when compared with preimmunisation and control animal data (Figure 1c, d and e).

Results support previous findings that VP2, NS1 and NS2 induced specific antibody responses in cattle, although the role that NS1 and NS2 antibodies play against BTV infection was not studied (Anderson et al. 2014). In a previous study, specific responses for two domains of VP2 encompassing amino acids 63–471 and 555–956 and for VP5 lacking the first 100 amino acids was observed (Mohd Jaafar et al. 2014); findings in this study added novel information of strong specific immune response to cVP2 encompassing 78 (position 337–415) amino acids. One limitation of this study was that, since the main objective here was to determine if the recombinant proteins were immunogenic, single dilution (1/200) was used in I-ELISA, and so the antibody levels were not titrated. Also, monitoring of antibody responses to the individual antigens in Group 4 mice injected with a mix of the three proteins would also have provided better insights into understanding the proteins’ dynamics when administered together.

### Cross-reactive neutralising antibody responses in mice

By Day 40 post-immunisation, all the mice (except control mice) showed detectable neutralising antibody (Nab) levels by serum neutralisation test (Table 2). Serum neutralisation test analysis revealed that anti-cVP2 (Group 1) antibodies could neutralise BTV-1, -2, -4, -9, -10, -16, -21 and -23. High Nab titres of log 3.1 were produced for BTV-4, -9, -10 and -16, followed by a titre of log 2.5 for BTV-21, titre of log 1.9 for BTV-2 and log 1.6 for BTV-1 and -23. No neutralisation was observed for BTV-5, -12 and -24. Anti-VP5 (Group 2) antibodies produced high neutralisation titres of log 3.1 for BTV-4, -9, -10, -16, -21 and -23, followed by titre of log 2.5 for BTV-2, log 1.3 for BTV-1 and no neutralisation of BTV-5, -12 and -24. Anti-NS1 (Group 3) had Nab titres of log 3.1 for BTV-4, -16, -21 and -23, followed by a titre of log 1.6 for BTV-9 and -10. Absence of neutralisation was seen for BTV-1, -2, -5, -12 and -24. Sera from Group 4 (cVP2+VP5+NS1) mice produced high neutralising titres of log 3.1 for BTV-4, -10 and -16, followed by a titre of log 2.5 for BTV-9, -21 and -23, titre of log 1.9 for BTV-2 and titre of log 1.3 for BTV-1. No neutralisation was observed for BTV-5, -12 and -24. Sera from animals vaccinated with the commercial inactivated vaccine (Group 5) were found to neutralise BTV-23 with titres of log 2.8; titre of log 2.5 for BTV-1 and -2; titre of log 2.2 for BTV-10, -12 and -16 and titre of log 1.9 for BTV-4 and -9. No neutralisation was observed for BTV-5, -21 and -24. Inhibition of virus-induced CPE was not observed in the sera of the control animals (immunised with Montanide^TM^ ISA 201 VG emulsion alone).

**TABLE 2:** Serum neutralisation test antibody titres expressed as log_10_ values.

Group	BTV-1	BTV-2	BTV-4	BTV-5	BTV-9	BTV-10	BTV-12	BTV-16	BTV-21	BTV-23	BTV-24
Group 1	1.6 ± 0.13	1.9 ± 0.46	3.1 ± 0.78	nn	3.1 ± 0.46	3.1 ± 0.53	nn	3.1 ± 0.48	2.5 ± 0.64	1.6 ± 0.41	nn
Group 2	1.3 ± 0.23	2.5 ± 0.65	3.1 ± 0.88	nn	3.1 ± 1.06	3.1 ± 0.86	nn	3.1 ± 0.54	3.1 ± 0.49	3.1 ± 0.98	nn
Group 3	nn	nn	3.1 ± 0.65	nn	1.6 ± 0.5	1.6 ± 0.63	nn	3.1 ± 0.95	3.1 ± 0.12	3.1 ± 0.14	nn
Group 4	1.3 ± 0.3	1.9 ± 0.14	3.1 ± 0.93	nn	2.5 ± 0.95	3.1 ± 0.43	nn	3.1 ± 0.75	2.5 ± 0.48	2.5 ± 0.18	nn
Group 5	2.5 ± 0.7	2.5 ± 0.29	1.9 ± 0.31	nn	1.9 ± 0.34	2.2 ± 0.38	2.2 ± 0.12	2.2 ± 0.29	nn	2.8 ± 0.17	nn
Group 6	nn ± 0.05	nn	nn	nn	nn	nn	nn	nn	nn	nn	nn

Note: Titres were expressed as log_10_ reciprocals of the highest positive serum dilution showing neutralisation with standard error values.

BTV, bluetongue virus; nn, no neutralisation.

Neutralising antibody titres have been shown to be an essential component of the protective immune response against BTV (Anderson et al. 2014; Huismans et al. 1987; Roy et al. 1990). The neutralising ability of cVP2 strengthens the hypothesis that the cVP2 is immunologically significant corroborating the *in silico* findings of B-cell epitopes in the conserved region (Shiva Jyothi et al. 2018), thereby making a way for an important line of further investigation. To the best of our knowledge, this study presents the first report of immunisation of the cVP2 segment. However, there are reports where individual or a combination of virus proteins have been evaluated as vaccines for BT. Recombinant VP2 and VP5 have been shown to elicit neutralising antibodies (with Nab titres of up to log 2.408) and protect sheep against homologous BTV challenge, but not a cross-reactive antibody response (Anderson et al. 2013, 2014; Huismans et al. 1987; Mohd Jaffar et al. 2014; Roy et al. 1990). In this study, the results indicate a cross-reactive potential of cVP2, but not against all the tested serotypes.

A maximum Nab titre of log 3.1 was observed in immunised mice. In general, the non-structural BTV proteins have predominantly been associated with cross-serotype cellular immune responses (Andrew et al. 1995). NS1, being one of the most conserved BTV proteins, was included in this study with the aim of detecting neutralising antibodies against BTV serotypes and to check for its role (if any) in eliciting cross-neutralisation between serotypes. Although its role in humoral immunity is not clearly known, NS1 has been shown to be a strong inducer of cytotoxic T lymphocytes (CTL) in sheep, inducing both homotypic and heterotypic T-cell responses (Anderson et al. 2013; Andrew et al. 1995). This could explain the neutralisation of fewer serotypes when compared with other groups and consistent low Nab titres in the group injected with NS1 in this study.

The major intention in including a group of mice injected with all the three recombinant proteins was to assess if there exists any additive effect in Nab production. Our findings indicate either an equivalent or less (even though not significant), but not higher, Nab production against any of the BTV serotypes tested. However, it cannot be ignored that the doses of recombinant proteins and the inactivated vaccine are not ideally comparable. Interestingly, in the group vaccinated with the commercial inactivated vaccine harbouring BTV serotypes BTV-1, -2, -10, -16 and -23, cross-neutralisation with BTV-4, -9 and -12 was noticed, albeit with low Nab titres. Further studies in the natural host may shed more light into the role of CTL responses and additive effects of CTL and humoral immune responses.

High Nab titres observed in this study with the recombinant proteins indicate the added advantage of the designed subunit vaccine’s potential adaptability over the commercial inactivated vaccine. The subunit vaccine using cVP2, VP5 and NS1 seems promising for further development as a BTV vaccine that is safe, broad spectrum and potentially DIVA compliant. The potentiality of these recombinant proteins should further be evaluated in natural host along with challenge studies for it to be ultimately used as a vaccine candidate.

## Conclusion

In this study, we present the stages in the development and evaluation of a novel designed recombinant subunit BTV vaccine model that is effective against multiple BTV serotypes. The central hypothesis of this study was that the conserved epitope of BTV VP2 protein, as part of a subunit vaccine, should confer broad-spectrum immunity against most of the available serotypes of BTV. Because the developed subunit vaccine with all three proteins was shown to induce a specific immune response to the administered proteins and could also neutralise a range of BTV serotypes, we believe that the recombinant BTV proteins cVP2, VP5 and NS1 are of potential significance. Additional investigations on the determinants of the cellular immune response of these proteins and immunogenicity studies in the natural host with subsequent BTV challenge will give better insights into the immune response generated by these vaccines.
